# Systematic review and meta-analysis: the efficiency of bacteriophages previously patented against pathogenic bacteria on food

**DOI:** 10.1186/s13643-023-02352-9

**Published:** 2023-10-28

**Authors:** Danitza Xiomara Romero-Calle, Vinicius Pereira de Santana, Raquel Guimarães Benevides, Maria Teresa Alvarez Aliaga, Craig Billington, Aristóteles Góes-Neto

**Affiliations:** 1 Postgraduate Program in Biotechnology, State University of Feira de Santana (UEFS), Av. Transnordestina S/N, Feira de Santana, BA 44036-900 Brazil; 2 Department of Biological Sciences, Feira de Santana State University (UEFS), Feira de Santana, BA 44036-900 Brazil; 3 Biotechnology Area, Institute of Pharmaco-Biochemical Research, Faculty of Pharmaceutical and Biochemical Sciences, Higher San Andres University, P.O. Box 3239, La Paz, Bolivia; 4 Health & Environment Group, Institute of Environmental Sciences and Research, PO Box 29-181, Christchurch, 8540 New Zealand

**Keywords:** Biocontrol, Bacteriophages, Foodborne disease, Patent, Safety

## Abstract

Food-borne diseases are a global public health issue with 1 in 10 people falling ill after eating contaminated food every year. In response, the food industry has implemented several new pathogen control strategies, such as biotechnological tools using the direct application of bacteriophages for biological control. We have undertaken a systematic review and meta-analysis that evaluated the efficiency of patented phages as a biological control for food-borne pathogens and determined the physical–chemical characteristics of the antimicrobial effect. Included and excluded criteria was developed. *Included criteria:* Phage patent files with an application in biological control on food and scientific articles and book chapters that used phages patented for food biological control. *Excluded criteria: P*atent documents, scientific articles, and book chapters that included phage therapy in humans, animals, and biological control on plants but did not have an application on food were not considered in our study. The systematic analysis identified 77 documents, 46 scientific articles, and 31 documents of patents and 23 articles was included in the meta-analysis. *Listeria monocytogenes* and *Salmonella* sp. comprised most of the targets identified in the screening, so that we focused on these strains to do the meta-analysis. There are a total of 383 and 192 experiments for *Listeria* and *Salmonella* phages for quantitative data analysis.

Indexing databases for the bibliographic search (Scopus, Web of Science (WoS) and PubMed (Medline) were addressed by an automated script written in Python 3 Python Core Team (2015) and deposited on GitHub (https://github.com/glenjasper).

A random-effects meta-analysis revealed (i) significant antimicrobial effect of *Listeria* phages in apple, apple juice, pear, and pear juice, (ii) significant antimicrobial effect of *Salmonella* phages in eggs, apple, and ready-to-eat chicken, (iii) no heterogeneity was identified in either meta-analysis, (iv) publication bias was detected for *Listeria* phages but not for *Salmonella* phages. (v) ListShield and Felix01 phages showed the best result for *Listeria* and *Salmonella* biological control, respectively, (vi) concentration of phage and bacteria, time and food had significant effect in the biological control of *Listeria*, (vii) temperature and time had a significant effect on the antimicrobial activity of *Salmonella* phages. The systematic review and meta-analyses to determine the efficiency of bacteriophages previously patented against pathogenic bacteria on dairy products, meat, fruits and vegetables. Besides, the discovering of key factors for efficacy, so that future applications of phage biotechnology in foods can be optimally deployed.

**Supplementary Information:**

The online version contains supplementary material available at 10.1186/s13643-023-02352-9.

## Introduction

Foodborne diseases are a significant public health issue, causing pressure on healthcare systems, lost productivity due to worker illness, and harm tourism and impact trade. For the foodborne diseases causing diarrhea, a disproportionate burden falls on children under 5 years old and those living in low- and middle-income countries. In addition to environmental contamination, pollution in water, soil, and air, food processing and unsafe food storage are factors in illness development (WHO, 2020) [1].

Outbreaks of listeriosis, salmonellosis, campylobacteriosis, hemorrhagic colitis, and hemolytic uremic syndrome are still commonly associated with the consumption of processed and raw foods. In designing effective interventions to mitigate these outbreaks, consequences such as antibiotic resistance, gut microbiota disturbances, and residual effects on human health and the environment must be avoided. The increasing popularity of more natural and organic foods, changing consumer preferences, and large-scale production of food animals are also driving the need for new interventions [2].

One alternative to control foodborne pathogens in foods is the use of bacteriophages (phages). Phages are viruses that infect bacteria and Archaea, which have no machinery for generating energy, and no ribosomes for making proteins. Phages are very specific in targeting and infecting the host bacterial or archaeal species [2, 3].

The therapeutic potential of phages was recognized immediately after the discovery by d’Herelle and Twort at the beginning of the 20th century [4, 5]. Nonetheless, after the discovery and successful application of antibiotics, phage therapy was virtually forgotten in the Western countries (Americas, Western, Europe); however, phage therapy was routinely carried out in the former Soviet Union and Eastern Europe [5].

Because of the rise of multidrug resistance in bacteria and the scarcity of new antibiotics in the drug development pipelines (WHO, 2020), the interest in phage therapy has been rekindled for use in human health, veterinary medicine, agriculture, aquaculture, and food safety [3, 6–8].

Several products based on phages have been approved as food processing aids: LISTEX (effective against *Listeria monocytogenes*), SALMONELLEX (effective against *Salmonella enterica*), both from the company Micreos and ListShield^TM^ (effective against *Listeria monocytogenes*), Ecoshield^TM^ (effective against *Escherichia coli*), and SalmoFresh^TM^ (effective against *Salmonella enterica*) from the company Intralytix.

Patent applications in the life sciences are the basis for the commercialization of new life science and healthcare-related technologies as well as the critical metric of innovation safety [9]. Herein, we describe a systematic review and meta-analyses in order to determine the efficiency of bacteriophages previously patented against pathogenic bacteria on dairy products, meat, fruits, and vegetables. Besides, the discovering of key factors for efficacy so that future applications of phage biotechnology in foods can be optimally deployed.

## Materials and methods

The systematic review and meta-analysis were conducted in five stages: planning, bibliographic search, initial selection, final selection, quality data selection and quantitative data selection, a summary of data, and results.

The scope of the study is limiting to recruit all the database about bacteriophages previously patented with application for biological control on food although there are several scientific research that use bacteriophages but are not patented for food biocontrol but we are not interested on it because we would like to know the availability to use patented bacteriophages. Besides, we were only interested in studies between 1945 to 2021 around the world because it is a global study.

**Fig. 1:**
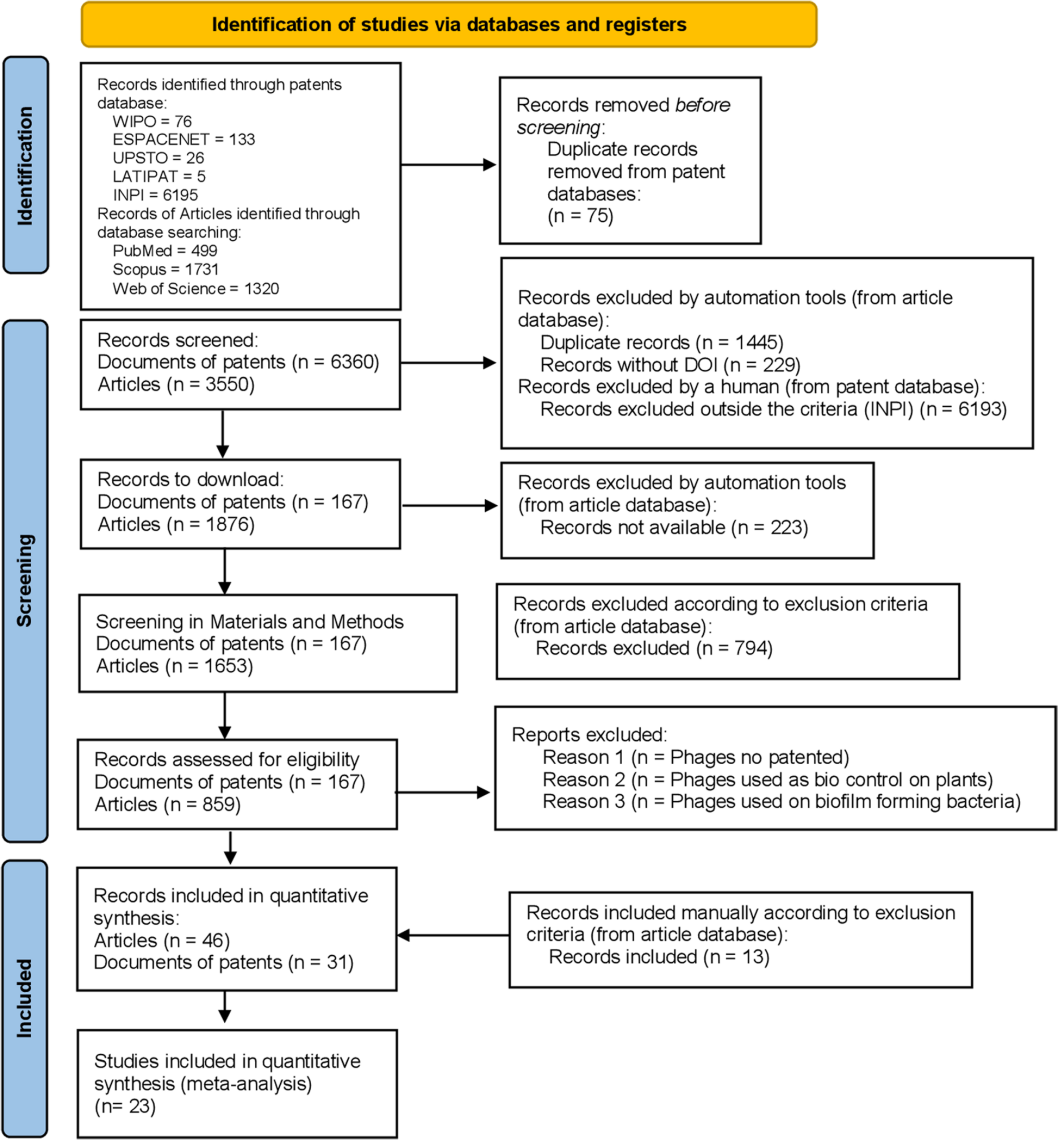
PRISMA work-flow applied to the study

All these steps were performed based on the bibliographic search protocols developed by Page et al. [10]. Indexing databases for the bibliographic search (Scopus, Web of Science (WoS) and PubMed (Medline) were addressed by an automated script written in Python 3 Python Core Team (2015) and deposited on GitHub (https://github.com/glenjasper). Subsequently, a manual review of outputs was performed by three independent reviewers (Fig. 1).

### Selection of articles and documents

#### First keyword screening: search in titles, abstracts and keywords sections

Scopus, WoS, and PubMed (Medline) databases were searched with the following search string: ((phage) OR (bacteriophage) OR (phage therapy) OR (biocontrol) OR (biosanitization) OR (biopreservation) AND (foodborne pathogens) OR (food safety)) in titles, abstracts, and keywords of the publications. Database searches included documents published since 1960 for Scopus, and from 1945 to October 2021, for WoS, it was not possible to use data restriction for PubMed.

The actualization of data was carried out in October 2021. The analysis of data was done from October 2021 to July 2022.

Databases were filtered, then the duplicate patent files were deleted, and unique documents were selected using a script (https://github.com/glenjasper/remove-duplicates, Table1 1s).

#### Second keyword screening: search in the materials and methods section

Keywords were searched in the materials and methods section of the publications with the following string: “phages, bacteriophages, biocontrol, the multiplicity of infection, MOI, PFU, *Streptococcus, Staphylococcus, Campylobacter, Shigella, Bacillus, Clostridium, Listeria, Salmonella, Enterobacter, Yersinia, Aeromonas, Pseudomonas, Escherichia*” using the script search_keywords.py (https://github.com/glenjasper/search-keywords).

#### Manual document review

A full-text manual review was conducted by three independent reviewers according to inclusion and exclusion criteria. During the extraction of quantitative data, 13 cited articles were added according excluded and included criteria (Table 2s).

### Selection of patent files

Patent databases (WIPO, ESPACENET, UPSTO, LATIPAT, and INPI) were used to identify the phage patents according to the following strings: WIPO *Keywords:* (phage OR bacteriophage) AND (biocontrol) AND (foodborne pathogens OR food safety) not (*Bacillus subtilis* or *Paenibacillus*). ESPACENET *Keywords:* (phages OR bacteriophages) AND (Food safety OR foodborne pathogens). USPTO *Keywords*: (Phages or Bacteriophages) AND (biocontrol) AND (foodborne), LATIPAT *Keywords*: (bacteriófagos) AND (alimentos) and INPI *Keywords*: (bacteriófagos) AND (alimentos).

The databases WIPO, ESPACENT, and UPSTO databases were filtered, then the duplicate patent files were deleted, and unique documents were selected using a script (a programming language for a special run-time environment that automates the execution of tasks, Table 3s). For LATIPAT and INPI databases, full-text manual reviews were conducted by three independent reviewers according to the inclusion and exclusion criteria.

Subsequently, the documents were selected according to exclusion and inclusion criteria, and all these processes were carried out by three independent reviewers (Table 4s).

Identification, screening, and included studies were carried out according PRISMA flow diagram guideline for systematic review and meta-analysis steps is described in Fig. 1.

### Selection criteria

Eligibility criteria were based on the PICO approach (*Population*: Food contaminated with foodborne pathogens, *exposure*: bacteriophage treatment or biological control, the *comparison group* the comparison group: Control: group food did not have the treatment with bacteriophages, and the experimental group: food that had the treatment with bacteriophages or biological control, and *outcome*: Bacteria log reduction log UFC/mL. Besides, study design, and date, certain features that were described in excluded criteria (Section 2.3.1) and included criteria (Section 2.3.2) and undertaken by three reviewers to avoid bias in the systematic review.

#### Included criteria

Bacteriophages previously patented for biological control on food described in patent documents, scientific articles, and book chapters that used phages patented for food biological control.

#### Excluded criteria

Bacteriophages without patent used for biological control on food described in patent documents, scientific articles, and book chapters that used phages patented for food biological control.

Bacteriophages previously patented for phage therapy in humans, animals, and biocontrol on plants but did not have an application on food were not considered in our study.

Studies (patents files and scientific articles) of phage biocontrol pathogenic bacteria biofilm on food, phages with chemical compounds, and those that did not evaluate the phage effect on the planktonic stage of pathogenic bacteria on food were also excluded. Moreover, unrelated, duplicated, unavailable full texts, or abstract papers were not considered for the study*.*

### Analysis of data and statistical analysis

The synthesis of the method: We did a systematic review using a pipeline and a manual screening using PRISMA method and for meta-analysis, articles were manually selected, random meta-analysis and meta-regression approach was developed, the heterogeneity and bias error of the study was analyzed.

Systematic review was developed, the keywords selection map was evaluated using Nvivo software (released in March 2020). For visualization of the data, VOSviewer® software was used, and the displayed network depicts the maps of authors and keywords (Van Eck and Waltman, 2009). VOSviewer combines visualization and clustering techniques, enhancing the analyses while bypassing unnecessary technical complications. This tool was designed for articles and chapter of book analyses, but not for patent documents.

Mapping of phage patents visualization was performed using Leaflet, an open-source JavaScript library for mobile-friendly interactive maps using the *script* (https://glenjasper.github.io/leaflet-phage-map/). This information was collected from patent documents and scientific articles.

Twenty-three scientific articles were selected for meta-analysis, the efficacy of *Salmonella* phages and *Listeria* phages on food (Tables 1 and 2, respectively). The efficacy and the heterogeneity of *Salmonella* phages and *Listeria* phages were evaluated by meta-analyses using (i) the in order to do the statistical synthesis, we used random-effect model because the assumption that the underlying true effects differ across trials (Bacteriophage antimicrobial activity could change be different using different foods) and (ii) standardized mean difference (SMD).

**Table 1:** Meta-analysis of food subgroups with *Listeria* phages

Food	*K*	SMD	95% CI	tau^2^	*I*^2^ (%)
Fresh sausage	05	−11.00	[−15.24; −05.70]	11.35	41.70
Apple	06	−00.27	[−01.30; 00.75]	00.82	43.70
Apple juice	03	−00.02	[−00.73; 00.68]	00.00	00.00
Cabbage	28	−236.16	[−299.82; −172.49]	11009.61	65.90
Catfish fillets	19	−179.82	[−237.25; −122.38]	12064.15	89.80
Cheese	112	−00.82	[−01.32; −00.30]	03.46	79.30
Chocolate milk	33	−201.00	[−256.79; −144.94]	5691.72	69.50
Honeydew melons	50	−500.00	[−59.87; −40.20]	197.9	92.80
Hot dogs	37	−313.00	[−365.19; −261.62]	00.00	00.00
Iceberg lettuce	08	−244.15	[−328.16; −160.13]	0.00	00.00
Lettuce	02	−75.00	[−134.20; −15.20]	620.15	25.40
Melon	03	−173.00	[−282.78; −63.70]	00.00	00.00
Melon juice	03	−307.14	[−403.05; −211.22]	00.00	00.00
Mixed seafood	12	−289.17	[−372.06; −206.28]	00.00	00.00
Mozzarella cheese brine	17	−377.34	[−468.33; −286.34]	00.00	00.00
Pear	03	−01.31	[−2.55; −0.07]	00.00	00.00
Pear juice	03	−0.06	[−0.76; 0.63]	00.00	00.00
Precooked sliced turkey	22	−07.57	[−11.33; −3.81]	20.04	85.50
Raw salmon fillet tissue	17	−208.63	[−280.39; −136.87]	7860.9	73.00
Red smear soft cheese	48	−333.36	[−384.34; −282.3]	00.00	00.00
Sliced cooked turkey breast	08	−138.70	[−187.83; −89.58]	00.00	00.00
Smoked salmon	14	−03.29	[−6.93; 00.35]	10.76	71.00
White mold soft cheese	24	−59.98	[−86.06; −33.90]	441.59	73.70

K = number of studies, *SMD* (Standardized mean difference; phage treated vs. control) and 95% CI (95 % confidence interval), tau^2^ = variance of the distribution of true effect sizes, *I*^2^ = test of heterogeneity / unaccounted variability. Effect of the direction (positive)

**Table 2:** Meta-analysis of food subgroups with *Salmonella* phages

Food	*K*	SMD	95% CI	tau^2^	*I*^2^ (%)
Apples	13	−00.38	[−1.42; 0.65]	02.21	81.30
Cantaloupe melons	03	−73.80	[−165.56; 17.95]	76.30	91.70
Chicken	03	−69.45	[−92.21; −46.69]	−69.45	00.00
Chicken breast	08	−5.52	[−07.65; −03.39]	05.08	61.90
Cooked meat	03	−97.89	[−136.77; −59.00]	-97.89	00.00
Eggs	02	−01.02	[−01.82; −00.21]	-01.02	00.00
Ground turkey	02	−654.71	[−916.89; −392.53]	00.00	00.00
Honeydew melons	12	−259.85	[−310.60; −209.09]	3336.40	45.00
Lettuce	17	−43.10	[−60.37; −25.83]	417.08	81.60
Milk	08	−70.63	[−104.24; −37.01]	1140.32	87.90
Mung bean sprouts	17	−164.34	[−197.91; −130.77]	2808.13	64.80
Pasteurized milk cheese	07	−309.94	[−423.93; −195.96]	13232.78	63.50
Pig Skin	22	−10.03	[−13.77; −6.28]	24.62	87.20
Precooked sliced turkey	26	−11.24	[−15.16; −7.33]	33.05	85.20
Raw cheese milk	02	−206.67	[−252.95; −160.38]	00.00	00.00
Raw meat	06	−26.78	[−37.57; −15.99]	00.00	00.00
Raw tuna	02	−114.39	[−160.84; −67.94]	00.00	00.00
Ready to cook chicken	03	−3.98	[−07.60; −00.37]	00.00	00.00
Romaine lettuce	23	−33.83	[−46.23; −21.43]	251.16	92.60
Sausage	08	−87.35	[−131.95; −42.75]	3183.30	93.40

K = number of studies, SMD (standardized mean difference; phage treated vs. control) and 95% CI (95 % confidence interval), tau^2^ = variance of the distribution of true effect sizes, *I*^2^ = test of heterogeneity / unaccounted variability. Effect of the direction (positive)

The effect size was calculated by default by Meta package. Furthermore, a principal components analysis (PCA) and meta-regression of some physical-chemical characteristics involved in the phage antimicrobial effect (temperature, time, initial phage concentration of phages, and bacteria) were also evaluated. ANOVA of the phages with respect to the log reduction of bacteria was measured. Begg and Mazumdar test: rank correlation test and funnel plot methods were used in order to determine bias error.

*The synthesis of results were carried out step by step in the beginning with the systematic review and meta-analyses as described in material and methods*.

## Results

### Systematic review

Identification of relevant documents relating to the use of phage for food biocontrol initially began by keyword searching of scientific articles using the terms described in materials and methods. This search identified 3550 records, including 499 records in PubMed, 1731 records in Scopus, and 1320 records in WoS. In total, 859 documents were identified. Subsequently, 45 documents in English and 1 in Polish were selected manually for further analysis by three independent reviewers and a fourth reviewer was involved as a tie breaker in case of disagreements. For patent screening, 6360 documents were identified in patent databases, 167 unique patents were chosen. In addition, 31 patents were manually selected by three reviewers (Fig. 1).

#### Spatial–temporal analysis of phage-based patents

**Fig. 2:**
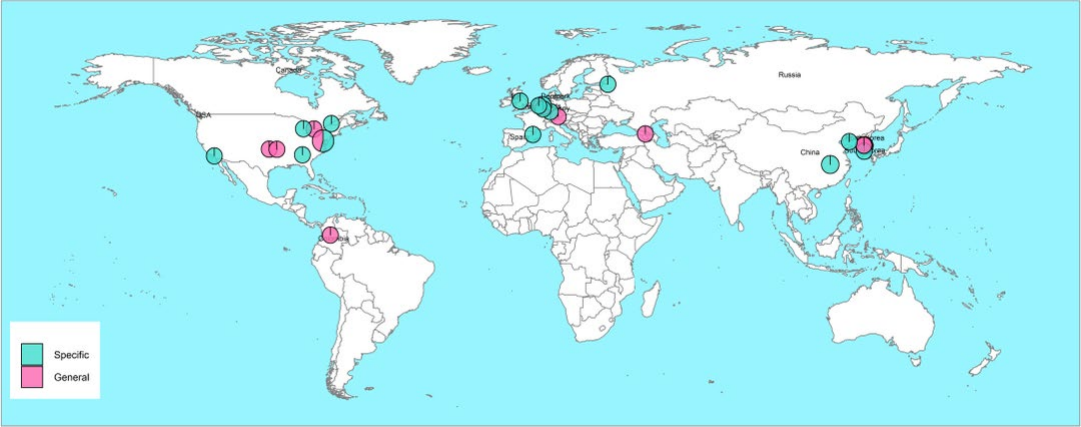
Map visualization of phage patents used for food biocontrol pink circles: for general food matrix, and sky-blue circles: for specific foods, the size of the circles represents the number of phage patents (https://glenjasper.github.io/leaflet-phage-map/)

A map visualization of applications for phage-based patents that made claims for phage biocontrol in foodborne diseases was made using Leaflet software (Fig. 2). The geographic distribution showed 41 phages patented applied on food, 46.34% from Europe, 29.27% from North America, 21.95% from Asia, and only 2.44 % from South America. There were no identified phage patents for food biological control in Africa and Oceania, (detail in https://glenjasper.github.io/leaflet-phage-map/). Overall, 58.54% of phage-based patents belonged to specific foods and 41.46% to general food (Fig. 2).

Regarding the temporal analysis, our study identified that the submission of phage-based patents with time revealed relatively infrequent patent activity from 1995 to 2006. The most applications in a single year occurred in 2007. After 2007, there was increased patent activity with several applications and publications of granted patents per year. Two applications and three publications of phage-based patents were retrieved in the last analyzed year (2019) (Supplementary material, Figure 1s). Although several phage-based patents were later withdrawn or not granted, an increasing interest in phages as antimicrobial agents in the food industry is evident.

#### Phage-based patent description for biocontrol usage

Among the 41 phage-based products patented for biocontrol in food, the minority of applicants (29.26%) were from universities and research centers, and the majority (70.74%) were from private companies. Target bacteria included *L. monocytogenes, Salmonella* sp., *E. coli, Pseudomonas* sp*., Shigella* sp., *Staphylococcus* sp*., Clostridium sp., Campylobacter* sp*.,* and *Staphylococcus* sp*.* The most frequent targets for biological control of foodborne diseases in patents were *L. monocytogenes* and *Salmonella* sp*.* Foods including dairy products, fruits, vegetables, meats, and fish were used as a matrix to test the biological control potential of the patented phage.

### Scientific article analysis

**Fig. 3:**
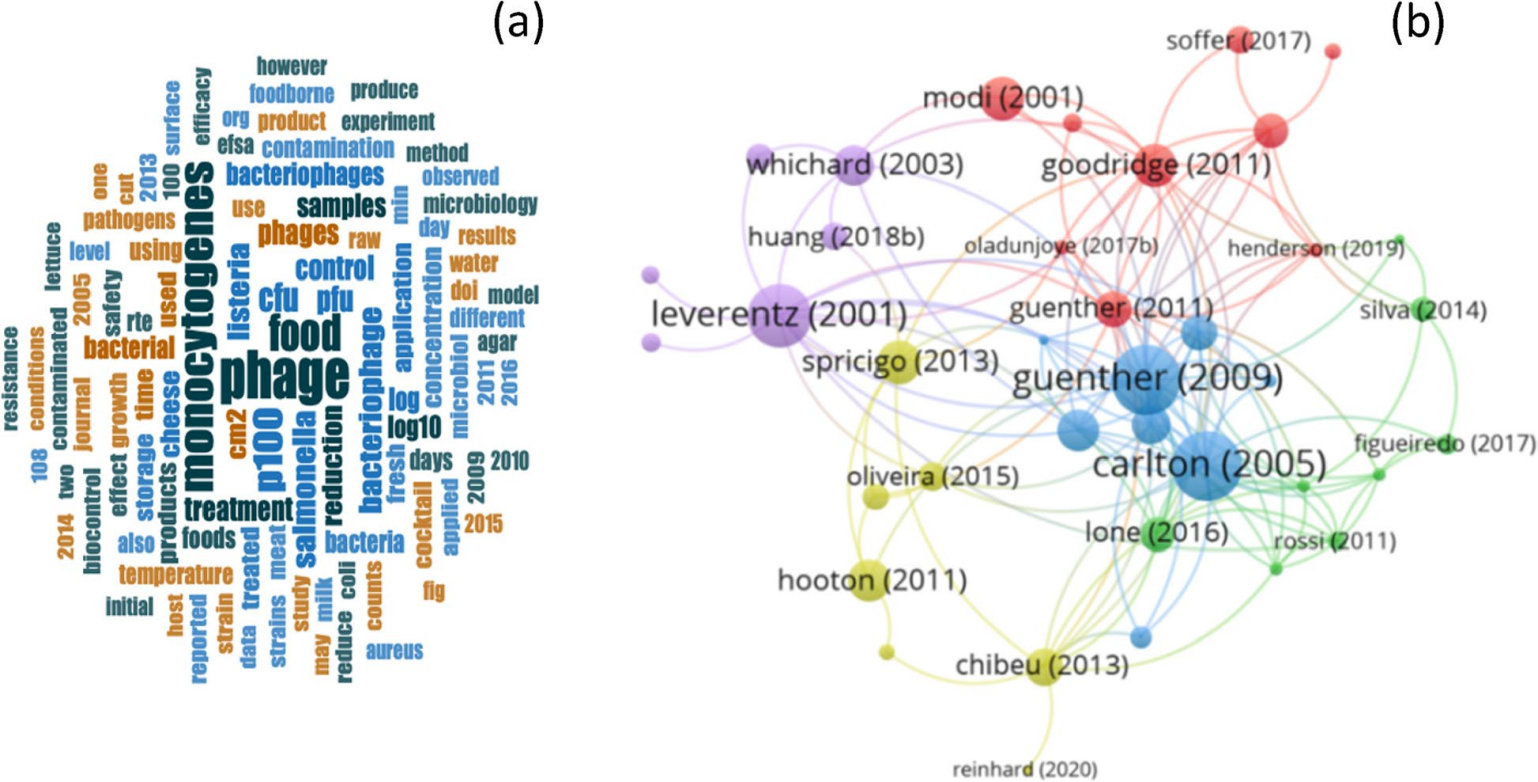
**a** Word map reflecting the most cited terms for all the evaluated articles used in the review processes. **b** Network visualization of association between the author and citations, each color represents a cluster

An analysis of the selected scientific articles was undertaken to determine the most frequent terms used in these food biocontrol studies. The results are shown as a word cloud (Fig. 3a). In total, 100 keywords were identified, including several words related to foodborne diseases. The most frequent keywords associated with this category were phage (1.26%) *Listeria monocytogenes* (0.94%), food (0.85%), P100 (0.73%), CFU (0.53%), bacteriophage (0.43%) *Salmonella* (0.42%), and others (94.84%).

The scientific article data were analyzed to identify the connections between the most relevant keywords in title and abstracts fields using the VOSviewer software. The association strength method was used for normalizing the strength of the links between items. As a result, five clusters were identified (Fig. 3b). The authors most cited in each of these clusters were Leverentz et al., [11], Guenter et al., [12], Goodridge [13] and Hooton et al., [14], and Lone [15].

### Meta-analysis

**Fig. 4:**
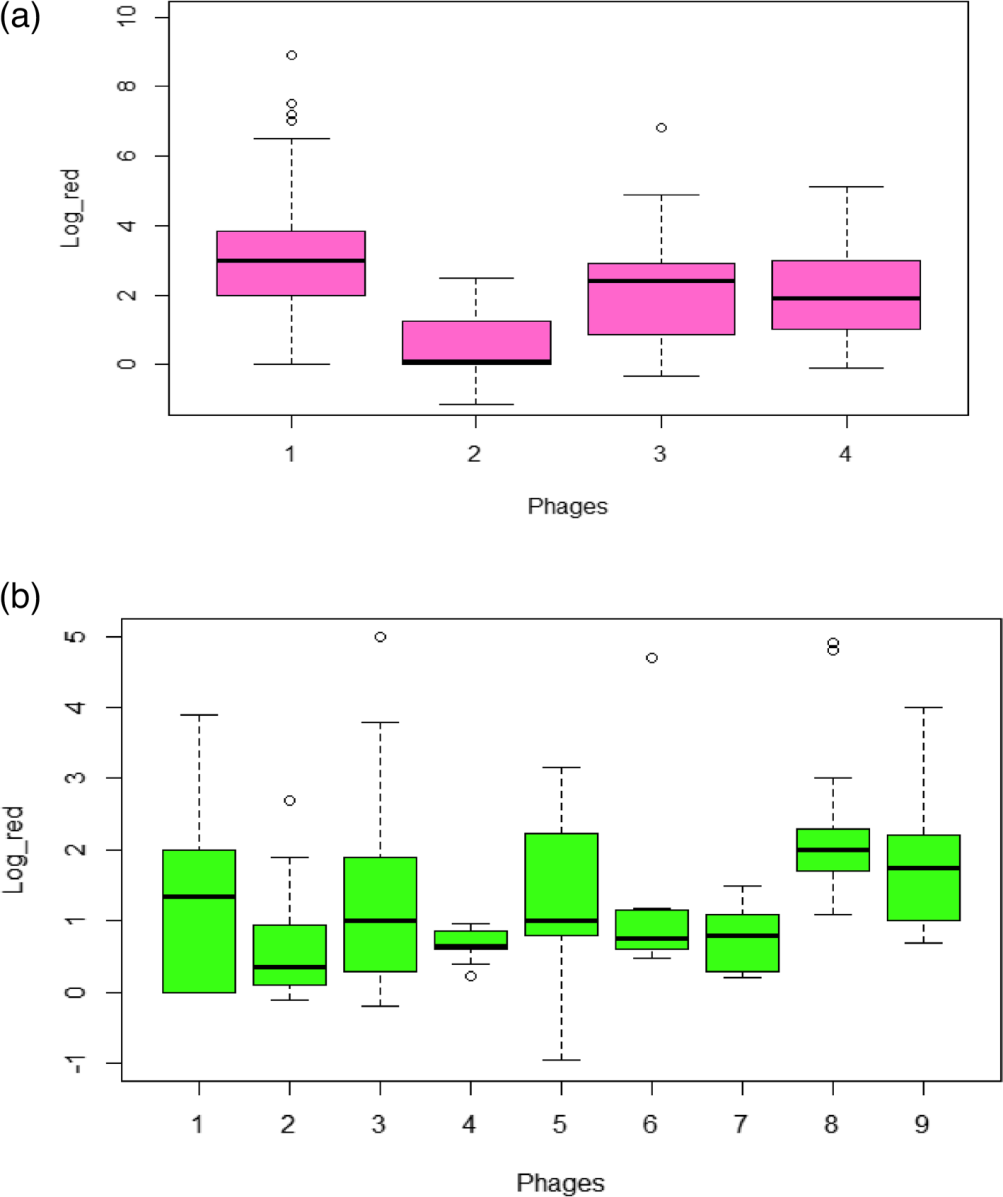
Boxplots of log_10_ CFU reductions of *Listeria monocytogenes* and *Salmonella* by different phages. **a** *Listeria* (1 = A511, 2 = ListShield, 3 = LM103 and LMP102, 4 = P100 and **b** *Salmonella* (1 = SCPLX1, 2 = Felix O1, 3 = LPST10, 4 = Felix O1, ФSH17, ФSH18 and ФSH19, 5 = SalmoFresh, 6 = SalmoLyse, 7 = P7, 8 = SJ2 and 9 = UABPhi20, UABPhi78, and UABPhi87)

The systematic analysis identified 77 documents, 46 scientific articles, and 31 documents of patents (Fig. 1), *Listeria monocytogenes* and *Salmonella* sp. comprised most of the targets identified in the screening, so that we focused on these strains to do the meta-analysis. A total of 383 and 192 experiments for *Listeria* and *Salmonella* phages for quantitative data analysis of these materials revealed *L. monocytogenes* and *Salmonella* sp. had a higher frequency of keywords in scientific articles (Fig. 4).

#### Listeria and Salmonella phage activity

To identify the antimicrobial activity of different phages, ANOVA of log reduction of bacteria and phages was determined. Four phages of *Listeria* (two single phages and two cocktails) and nine *Salmonella* phages (five single phages and four cocktail) were identified. *Listeria* and *Salmonella* phage data showed non-parametric distribution (Shannon index, *p*-value = < 2.2 × 10^−16^ and 4.53 × 10^−9^, respectively). Furthermore, significant differences in bacteria log reduction achieved were identified for *Listeria* and *Salmonella* phages (Kruskal–Wallis, *p*-value = < 2.2 × 10^−16^ and 2.67 × 10^−6^). ListShield™ phages had the lowest median log reduction of *Listeria* on foods (0.10log_10_ CFU/sample)*,* whereas the A511 phage had the highest median reduction (2.7log_10_ CFU/sample; Fig. 4a). Furthermore, A511, LM 103 and LMP 102 cocktail, and A511 phages showed outliers (Fig. 4a). For *Salmonella* applications, Felix 01 phage showed the lowest median log reduction of bacteria on foods (0.35log_10_ CFU/sample), and SJ2 had the highest median reductions (2.0log_10_ CFU/sample, Fig. 4b). Felix 01 phage, LPST10 phage, SalmoFresh, SalmoLyse, and SJ2 phage showed outliers (Fig. 4b).

#### Principal component analyses

**Fig. 5:**
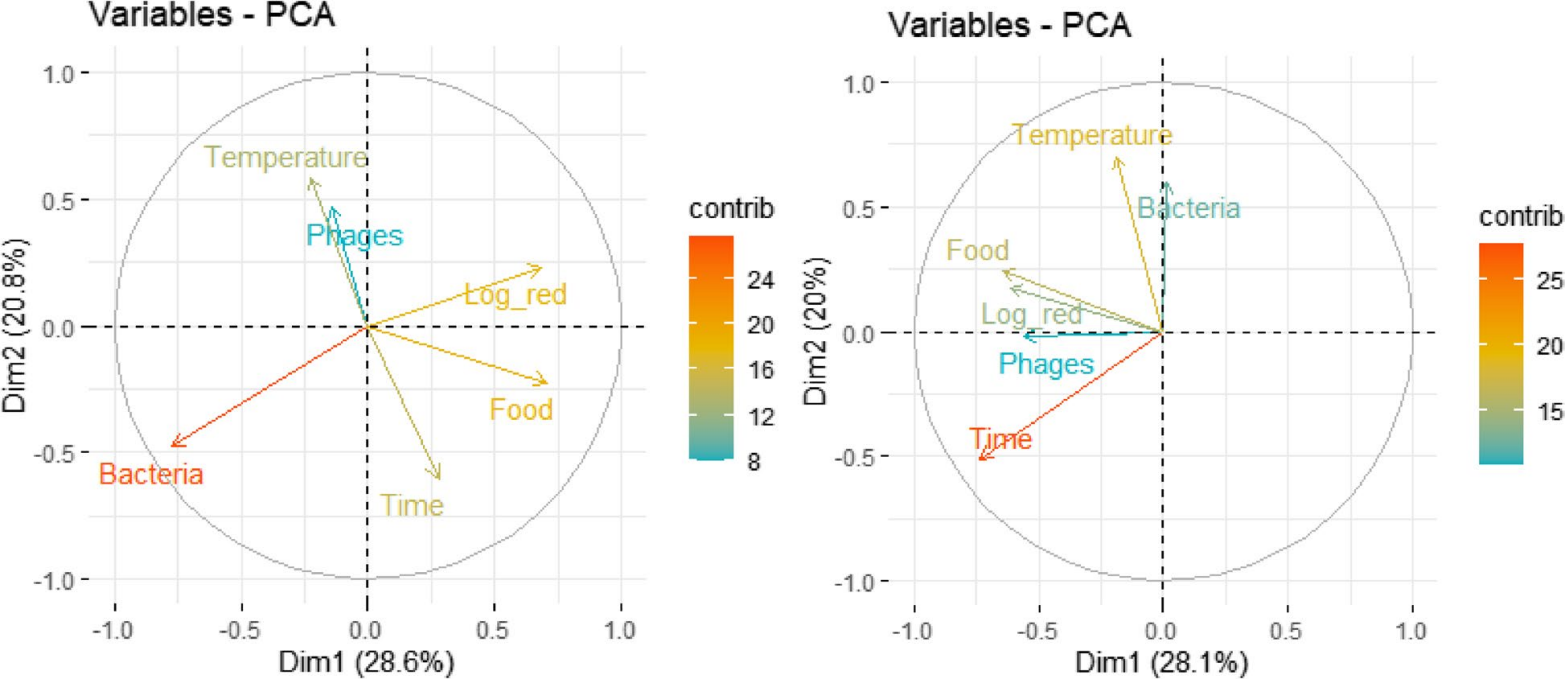
Principal components analysis on food biocontrol variables for *Listeria* and *Salmonella* phages. **a** *Listeria* phages PCA (49.4%) and **b** *Salmonella* phages PCA (48.1%)

Principal components analysis (PCA) was carried out for *Listeria* and *Salmonella* phage biocontrol variables (Fig. 5). For *Listeria* phages, phage concentration was positively associated with temperature in Principal Component 1 (PC1) and bacteria log reduction is associated positively with food in Principal Component 2 (PC2), with bacteria log reduction having the highest contribution to the PCA (Fig. 5a). For *Salmonella* phages, bacteria concentration was positively associated with temperature in PC1, and bacteria log reduction was associated positively with food, the concentration of phages and time in PC2, and time had the highest contribution to PCA (Fig. 5b).

#### Meta-regression of physicochemical parameters

Significant correlation was identified between the log reductions of *Listeria* and initial concentration of phages (*p*-value = 1.05 × 10^−5^), initial concentration of bacteria (*p*-value ≤ 2 × 10^−16^), time (*p*-value = 1.44 × 10^−9^), and food type (*p*-value = 8.16 × 10^−5^). An overall positive and significant correlation was found (adjusted R-square = 0.2764, *p*-value = 2 × 10^−16^, intercept = 7.69) for the described physical-chemical factors and log reduction by *Listeria* phages.

For *Salmonella* phages, temperature (*p*-value = 0.00825) and time (*p*-value = 0.00374) showed positive and significant correlation with log reduction of bacteria. But, overall, there was no significant correlation for physical–chemical factors with log reduction bacteria (adjusted R-square = 0.09095, *p*-value = 0.0003457, intercept = 0.46).

#### Meta-analysis of Listeria and Salmonella phages in different food matrices

We investigated the effect of the food matrix on the biological control efficacy of *Listeria* and *Salmonella* phages. A meta-analysis of the antimicrobial effect of *Listeria* phages on vegetables, meat, and dairy products was undertaken on 383 experiments from the literature. In these experiments, a significant antimicrobial effect was found (*p*-value < 0.0001); however, there was high heterogeneity (*I*^2^ = 82.0% [80.5%; 83.4%] and tau^2^ = 12.94 [<0.00; <0.00]). Further analysis of the standardized mean difference (SMD = −3.15; 95% CI= −3.90 and −2.40) revealed there was a significant antimicrobial activity effect of *Listeria* phages (a lower bacteria concentration in the treated group) and a high effect size (*z* = −8.24), and a positive effect, positive trends were reported for bacteriophages as biological control on food.

To better understand these data, a subgroup meta-analysis was undertaken for 23 foods individually (Table 1). There were significant differences found both between and within groups (*p*-value < 0.0001). The strongest effect of *Listeria* phages was found in Mozzarella cheese brine (SMD = −377.34) and the weakest effect of the phages was found in apple juice (SMD = −0.02). Results from experiments with hot dogs, apple juice, iceberg lettuce, melon, melon juice, mixed seafood, mozzarella cheese brine, pear, pear juice, red smear soft cheese, and sliced cooked turkey breast showed no heterogeneity (0 for *I*^2^ and tau^2^). Fresh sausage, apple, and other lettuce experiments showed moderate heterogeneity (*I*^2^ < 50). Experiments with cabbage, catfish fillets, cheese, chocolate milk, honeydew melons, precooked sliced turkey, raw salmon fillet tissue, smoked salmon, and white mold soft cheese displayed high heterogeneity (*I*^2^ < 75) (Table 1).

A meta-analysis of the antimicrobial effect of *Salmonella* phages on vegetables, meat, and dairy products was undertaken using 192 experiments. In these experiments, a significant antimicrobial effect was found (*p*-value < 0.0001), again with high indexes of heterogeneity (*I*^2^ = 89.6% [88.4%; 90.7%] and tau^2^ = 30.4226 [1131.28; 2752.20]). There was a significant antimicrobial activity effect of *Salmonella* phages when comparing the standardized mean difference of treated and untreated groups (SMD= −11.21; 95% CI= −12.79 and −9.62) and a high effect size (*z* = −13.89). As well as with *Listeria* phages, a subgroup analysis was undertaken to explore the effect of *Salmonella* phages on each different food type (Table 2). Positive trends were reported for bacteriophages as biological control on food.

Nineteen foods were analyzed as subgroups, and significant differences between and within groups were detected (*p* < 0.0001). The biggest effect of *Salmonella* phages was found in ground turkey (SMD = −654.71) and the least effect of the phages was found in apples (SMD = −0.38). Experiments with chicken, cooked meat, eggs, ground turkey, raw cheese milk, raw meat, raw tuna, and ready to cook chicken showed no heterogeneity (*I*^2^ = 0). Honeydew melon experiments showed moderate heterogeneity (*I*^2^ < 50). Experiments with apples, cantaloupe melons, chicken breast, lettuce, milk, mung bean sprouts, pasteurized milk cheese, pigskin, precooked sliced turkey, romaine lettuce, and sausage exhibited higher heterogeneity (*I*^2^ < 75; Table 2).

#### Bias error detection of meta-analysis of Listeria and Salmonella phages

**Fig. 6:**
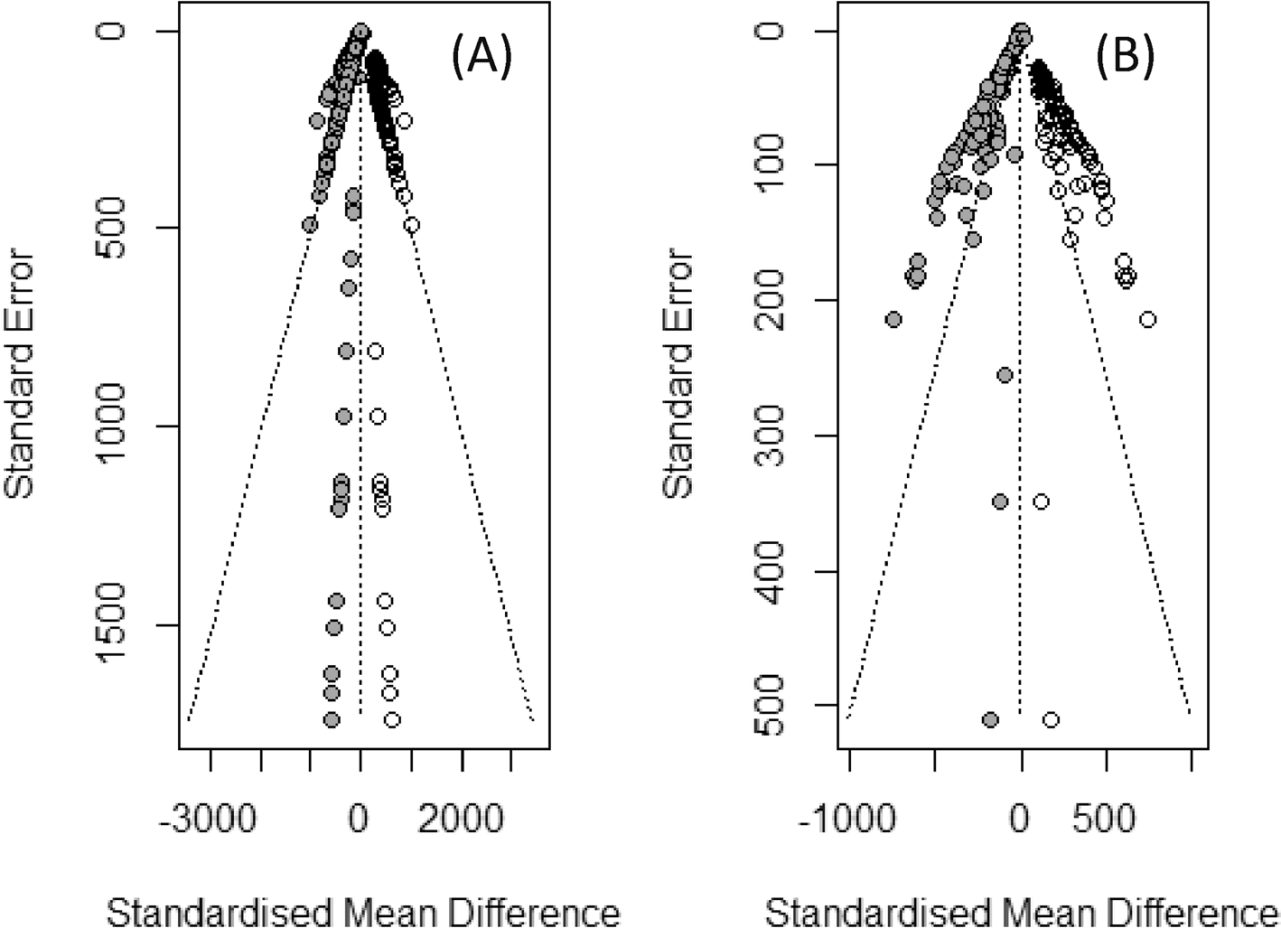
Funnel plot of antimicrobial effect of *Listeria* and *Salmonella* phage on foods. **a** Effect of *Listeria* phages and **b** *Salmonella* phages

A regression test using funnel plot asymmetry showed significant systematic error (*p*-value = 0.0003643), and high size effect (*z* = 3.5) for *Listeria* phage patents, and no significant error was detected in *Salmonella* phages patents (*p*-value = 0.58), as well as low effect size (*z* = 0.55) (Fig. 6).

## Discussion

The foodborne diseases are a public health problem, alternatives to antibiotic against pathogenic bacteria as bacteriophages for biocontrol needs to be studied and applied on food. There is scarce literature on using systematic review and meta-analysis methods to evaluate and improve the application of interventions for foodborne pathogens, such as phage biocontrol. The systematic review and meta-analyses of phage applications in food could be important tools to evaluate the efficiency of the phages previously patented on biological control for food, the physicochemical compounds that play a crucial role in the antimicrobial activity.

On the other hand, the geographical global distribution of both of the patent documents and scientific articles on phage biocontrol in foods reported in this work was mainly (>80%) in North America and Europe, with Asia and South America only minor contributors (detail in https://glenjasper.github.io/leaflet-phage-map/). This contrasts with patent applications for other food sectors, such as food crops, whose patents are predominantly (43%) filed in Asia [5]. This may be explained by the longer association of phage research with European and North American laboratories dating back to the works of Twort (UK) and d’Herelle (France/Canada) at the turn of the 19th century.

The patent review showed sporadic filings from the late 1990s and then a notable increase in 2007, followed by the publication of these filings in 2009 and 2010. This 3-year period coincides with the first regulatory approvals and release of phage products to the market for foodborne pathogen biocontrol of *Listeria* [16]. From then on, applications and publication of phage patents for food use have been increasing steadily. This trend of patent applications has also been reported for the use of phages in crop plant protection [16].

Besides, most patent applicants for biocontrol in foods (73.18 %) were from private companies, and the minority of applicants was from universities and research centers (26.82%). Intralytix Inc. has more patents than any other company with 41% of the total patents in this field. In contrast, patents for phage biocontrol of plant pathogens have been mostly filed by academia (56%), with a minority (37%) linked to industry (without joint applicants), and 7% were joint applicants [5].

In recent years, several studies have been published for biocontrol of phages in bacterial plant pathogens, such as *Dickeya, Ralstonia*, *Xanthomonas*, and *Pseudomonas*, with promising results [5]. Although the infection properties of a given phage may appear to have great potential with in vitro studies, this does not necessarily is translated into biocontrol potential in the field, so that field or greenhouse trials are very important for this research type. Nonetheless, in the food industry, there may be greater potential for phage biocontrol: from the decontamination of livestock to the sanitation of equipment and contact surfaces on farms and industry [13]. This could be a reason for increased commercial interest in phages for food safety instead of crops.

Regarding to the network analysis of these keywords revealed the more cited article was that of Guenther et. al [12], studying *Listeria* phage P100, which is one of the most widely studied phages and was the key active ingredient in the first phage product (Listex^TM^ P100) approved by the USDA (GRAS notice GRN 000198) for use in foods. The European Food Safety Authority has also evaluated the safety and efficacy of Listex^TM^ P100 during the processing of three ready-to-eat (RTE) product categories (meat and poultry, fish and shellfish, and dairy products) [17–19].

In this study, we identified that bacteriophages previously patented against *Listeria* and *Salmonella* biological control for food showed a significant efficiency and positive effect, so it could be an alternative as antibiotic care for foods as dairy products, meat, fruits and vegetables. No heterogeneity was identified, so we present a homogeneous data.

In several studies using bacteriophages for biocontrol of *L. monocytogenes* in food products, such as raw meat, smoked fish, fermented fish, milk, cheeses, fresh-cut fruits, vegetables, and various ready to eat products, authors succeeded with the reduction or even eradication of *L. monocytogenes* from food products. Where most of these trials were performed with P100 phage, then PhageGuard Listex cocktail bacteriophage, ListShield cocktail bacteriophage, and only a few attempts were performed with other bacteriophages [20].

In our meta-analysis of *Listeria* phages, ListShield™ phages had the best performance for biocontrol for *L. monocytogenes* in apples, cheese, lettuce, and smoked salmon. ListShield*™* (formerly LMP-102) is produced by Intralytix Inc and is a cocktail of six distinct lytic phages: LIST-36 (ATCC # PTA-5376), LMSP-25 (ATCC # PTA-8353), LMTA-34 (ATCC # PTA-8354), LMTA-57 (ATCC # PTA-8355), LMTA-94 (ATCC # PTA-8356), and LMTA-148 (ATCC # PTA-8357) [21], and for Salmonella phages, Felix O1 exhibited the best antimicrobial effect on food among other bacteriophages [22–25].

When examining the methods of phage application in the selected articles, phage cocktails were used in 50% of the *Listeria* studies and 44.44% of the *Salmonella* studies. For most of the applications, cocktails of phages are likely required to achieve good coverage of all strains as most phages are intrinsically narrow in host range [26]; however, there are some exceptions, such as P100, which can infect ~95% of *L. monocytogenes* strains in serovars 1/2 and 4 [12].

With respect to the physicochemical parameters, the initial concentration of phages and bacteria, time of storage, and food type had a significant correlation with the log reduction of bacteria for *Listeria* phages. In general, increasing the initial phage: host ratio has been found to enhance the efficacy of the phage in reducing bacterial populations [12, 22, 27–36]. Guenther et al. [12] suggested that phages suspended in liquid foods can diffuse almost freely, and thus, their distribution and potential contact with their host cells does not appear to be a problem. Nevertheless, on solid foods, such as hot dogs, salad leaves, and other produce which have an uneven surface, and where the surface properties or total surface area accessibility are limited, the parameters may be of great importance. For *Salmonella* phage treatments, temperature and time also had a significant effect.

The meta-analysis showed that phages specific for foodborne pathogens *Salmonella* spp. and *L. monocytogenes* significantly reduced pathogens on food, but high heterogeneity was detected. This heterogeneity could be explained by subgroup analyses of individual food types in both cases. Sabitova et al. [37] reported that a meta-analysis ideally combines the results of several studies that are highly comparable in design, intervention, and patient population. However, in real life, meta-analyses frequently contain multiple, relatively small studies that differ in many respects [37]; hence subgroup analysis is warranted. When examining 23 food subgroups tested with *Listeria* phages, 11 subgroups reduced the heterogeneity to 0, and 3 subgroups reduced the heterogeneity to moderate, which represents 60% of studies. It was notable that subgroups with number of samples higher than 14 displayed more heterogeneity. Similarly, to *Listeria* phages, in *Salmonella* phages, the heterogeneity was reduced among the 20 food subgroups, 8 reduced to 0, 1 to moderate, so that 45% of the subgroups had reduced heterogeneity. As the *Listeria* phages subgroups, where the number of samples was higher than 13, showed more heterogeneity. The increasing data heterogeneity with increasing sample number for both groups of phage experiments is likely due to the natural physical heterogeneity of food products when tested across studies undertaken in different countries, climates, and with different varieties of foods.

For the smaller subgroup analyses, it may not be possible to estimate heterogeneity with much precision as *I*^2^ has a substantial bias when the number of studies is small [38]. In small meta-analyses, confidence intervals should supplement or replace the point estimate *I*^2^ [38].

This study showed the effectiveness of bacteriophages previously patented as biological control on food, the potential use for biotechnological applications in food industry, increase the use of bacteriophages, promote the laws and regulations for its use, future research about the use of this strategy on different foods, pathogenic bacteria, and other countries.

The limitation of the study was that there were not much studies about the use of bacteriophages previously patented using for food bio control.

## Conclusions

In summary, we evaluated the efficiency of phage-based patents as a biological control for fruits, vegetables, and meat. Our meta-analyses revealed that initial concentration of phage and bacteria, time, and food were associated with an antimicrobial effect on *Listeria.* Temperature and time were associated with an antimicrobial effect on *Salmonella*. ListShield and Felix01 phages showed the best result for *Listeria* and *Salmonella* biological control, respectively.

The use of phages has much promise to control bacterial pathogens in food industries and other applications. It is evident that the application of phages to each food system and pathogen needs to be optimized and that some food matrices are more challenging for phage use than others. A systematic approach, such as we have used herein, will help inform future applications of phages to foodborne bacterial pathogens and highlights the need to improve the comparability of results to give the best confidence in the conclusions of such studies.

### Supplementary Information

**Additional file 1: Table 1s.**

**Additional file 2: Table 2s.**

**Additional file 3: Table 3s.**

**Additional file 4: Table 4s.**

**Additional file 5: Fig 1s.** Number of phage for food biocontrol patents with respect to application and publication dates.

## Data Availability

The database provide in this article is available to use.
