# Supplementary figures and images for: Systematic review and meta-analysis: the efficiency of bacteriophages previously patented against pathogenic bacteria on food
Source: Syst Rev. 2023 Oct 28;12:201. doi: 10.1186/s13643-023-02352-9 (PMC10612260; doi:10.1186/s13643-023-02352-9)

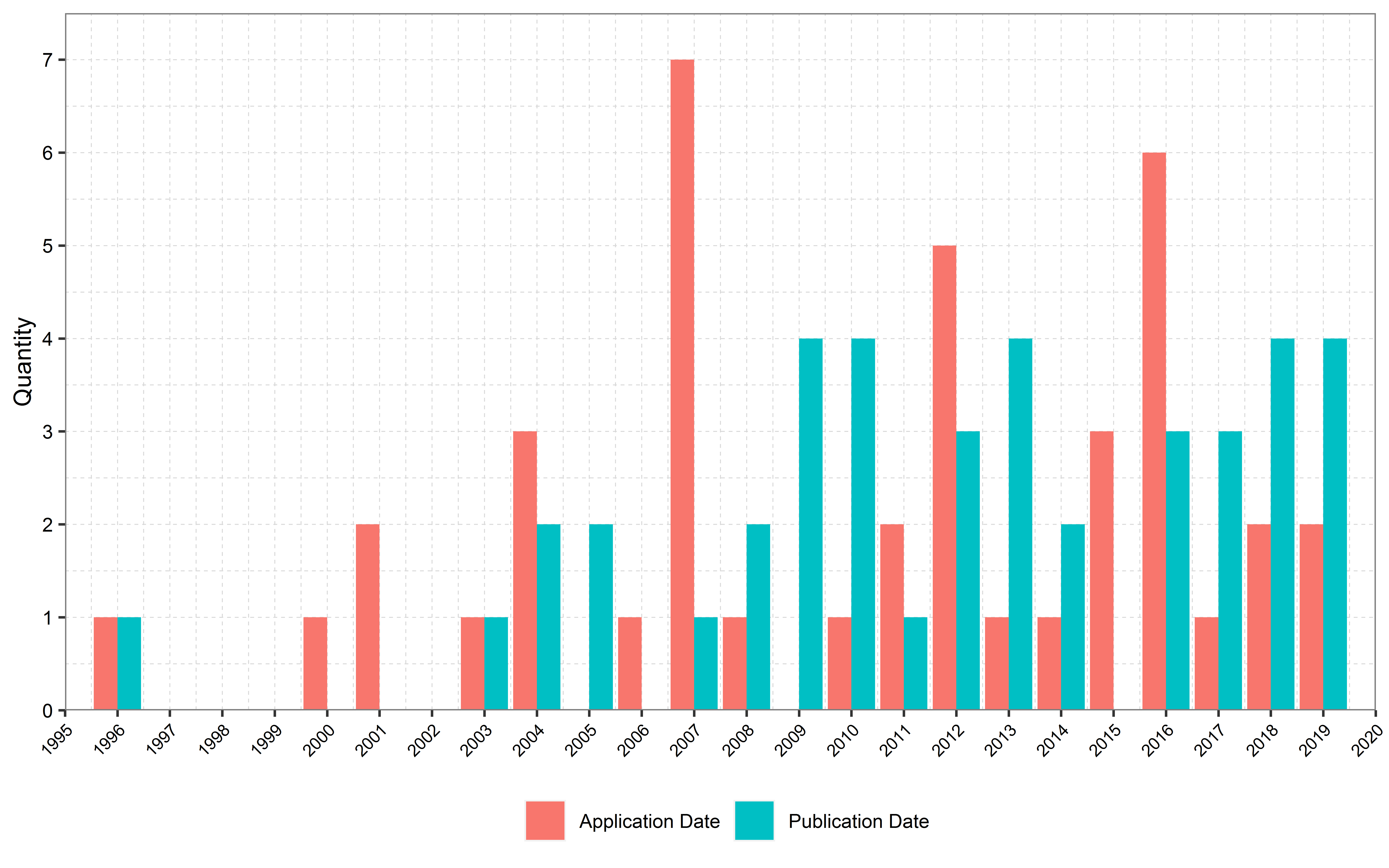

Supplement: Supplementary file 5 — Additional file 5: Fig 1s. Number of phage for food biocontrol patents with respect to application and publication dates. [file 13643_2023_2352_MOESM5_ESM.tiff]
